# Construction of Sensory/Mass Spectrometry Feedback Platform for Seeking Aroma Contributors during the Aroma Enhancement of Congou Black Tea

**DOI:** 10.3390/plants11060823

**Published:** 2022-03-20

**Authors:** Sifan Mei, Yanyan Cao, Gang Zhang, Su Zhou, Yi Wang, Shuying Gong, Qiang Chu, Ping Chen

**Affiliations:** 1 Tea Research Institute, Zhejiang University, Hangzhou 310058, China; meisifan@zju.edu.cn (S.M.); yycao@zju.edu.cn (Y.C.); 22016202@zju.edu.cn (G.Z.); 3180102132@zju.edu.cn (S.Z.); shuygong@zju.edu.cn (S.G.); 2 Department of Food Science, Zhejiang University, Hangzhou 310058, China; 3 School of Humanities and Education, Chongqing City Management College, Chongqing 401331, China; mindy9513@163.com

**Keywords:** Yunnan congou black tea, aroma enhancement, aroma attributes, volatile compounds, multiple factor analysis

## Abstract

Baking is widely accepted for aroma enhancement of black tea, and studies have mainly focused on the aroma or chemical substances under a specified baking condition. Understanding of the feedback between aroma substances and characteristics is urgently needed. Therefore, a mutual feedback platform (SES/MS) combined sensory evaluation system (SES) with gas chromatography–mass spectrometry (GC-MS) was established. Based on this platform, we found that baking at 90 °C for 4 h or 5 h could maintain the primary aroma attributes and increase characteristic aroma attributes—these were considered to be the best baking conditions for Yunnan congou black tea. Meanwhile, 47 volatiles were identified, among which, pyrrole and benzaldehyde appeared to have a caramel aroma, and 2-furanmethanol and α-terpineol presented a baked aroma. This study reveals the dynamic change of aroma profiles and compounds during the aroma enhancement, and provides an optional template for researchers, focused on the relationship between quality and aroma attributes of teas.

## 1. Introduction

The annual output and consumption of black tea rank first in the world, which is partly attributable to the fragrant, charming, and elegant aromas generated during the initial process [1]. To obtain enhanced aroma and taste, baking technology has commonly been applied [2]. Studies had shown that baking could improve the aroma and taste of both oolong and yellow tea. The slow-bake process can give oolong tea a full-bodied flavor, with caramel-flavored and mellow-scented characteristics [3]. With increase in baking temperature and time, the aromas of oolong tea gradually change from stuffy, green, and miscellaneous aromas to pure, fresh, sweet, and caramel aromas [4]. By establishing a thermal reaction model of D-glucose and L-theanine, Guo et al. [5] found that 2,5-dimethyl pyrazine, formed during the full-firing process, is the key ingredient of the “roasted peanutty” flavor of Wuyi rock tea. Likewise, roasting is considered to be critical in forming the “crispy-rice-like” flavor of large-leaf yellow tea [6]. These studies indicate that proper baking and aroma enhancement treatment could amplify the intrinsic aromas and produce characteristic flavors, benefitting from the type, concentration, and proportion variations of volatile components.

Nevertheless, research involving the aroma of black tea mainly pay attention to the fermentation process [7], the main processing technology of black tea, as well as the region [8] and grade [9,10]. Homogeneously, studies have simply focused on the variation of chemical substances under a specified baking condition, and have not provided a scientific sensory evaluation of roasted black tea. Qiu et al. [11] found that the relative content of volatile alcohols, aldehydes, esters, and ketones increased in Dianhong after baking. Yang et al. [12] showed that 1–1.5 h of baking at 70 °C or 90 °C could indeed improve the content of benzyl alcohol, phenethyl alcohol, etc., in black tea. There are correlation analyses based on fragrance perceptions and aroma active chemicals, which were applied for distinguishing different grades of Dianhong samples [13]. It is necessary to establish an understanding of the mutual feedback between aroma substances and aroma characteristics during the baking process of black tea, which could, in turn, provide guidance for the processing technology.

Therefore, visibly dynamic changes of aromas and substances during the baking process are vital for the quality of black tea. There is an urgent demand for a mutual feedback platform, utilizing the sensory evaluation data to guide the seeking of beneficial aroma substances, which in turn can offer a theoretical basis for the improved aromas and processing technologies. In this study, a scientific sensory evaluation system (SES) was established, and was applied for an original quality evaluation. A modified simultaneous distillation extraction (SDE) method and gas chromatography–mass spectrometry (GC-MS) were applied for aroma extraction and identification. In addition, multiple factor analysis (MFA) was used to present the relationship between tea samples, aroma attributes, and aroma substances in a graphical overlay, so as to investigate the aroma quality and characteristic flavor contributors of aroma-enhanced Yunnan congou black tea. Based on the feedback platform between SES and GC-MS, we aimed to explain the material basis for the improved aromas during the baking process and offer guidance for the configuration of the processing parameters for black tea. Meanwhile, the feedback platform constructed in our study could provide a template for the promotion of quality in teas.

## 2. Material and Methods

### 2.1. Samples and Chemicals

Tea samples were made from the “Yunnan Dayezhong” variety (*Camellia sinensis var. assamica*) by Yunnan Dianhong Group Co., Ltd. (Yunnan, China), according to the traditional method, in October 2020. The finished black tea (Dianhong—DH) was baked in a hot-air oven (Shanghai Dianjiu Chinese Machinery Manufacturing Co., Ltd., Shanghai, China) at 80 °C, 90 °C, and 100 °C for 0–7 h, separately. Tea samples were taken out at 1 h intervals, sealed with opaque aluminum foil bags, and stored at −20 °C until used.

Ultrapure water was made by a Synergy UV water purification system (Millipore Sas, Hardt Molsheim, France). Anhydrous ether and acetone of analytical grade were purchased from Sinopharm Reagent Group (Shanghai, China). Reference chemical standards (Vanillin, 2,5-dimethylpyrazine, 4-hydroxy-2,5-2methyl-3-2(H)furanone, ethyl acetate, and guaiacol) for quantitative descriptive analysis (QDA) were obtained from Aladdin Reagent Co., Ltd. (Shanghai, China). Authentic aroma standards (Linalool, methyl salicylate, α-terpineol, styrene, benzyl alcohol) for substance identification were bought from Yuanye Biological Technology Co., Ltd. (Shanghai, China). Decanal, 2-furanmethanol, 1-ethyl-1H-pyrrole-2-carbaldehyde, 2-acetylpyrrole, 2,5-dimethyl-pyrazine, and geraniol were purchased from TCI Development Co., Ltd. (Shanghai, China). Cis-2-penten-1-ol, pyrrole, benzaldehyde, geraniol, indole, benzene ethanol, hexanal, and furfural were bought from Aladdin Reagent Co., Ltd. (Shanghai, China). Phenylacetaldehyde, 2,6,6-trimethyl-1,3-cyclohexadiene-1-carbaldehyde, and trans-2-hexenol were purchased from Macleans Biochemical Technology Co., Ltd. (Shanghai, China). Anhydrous ethanol and α-ionone were obtained from Sigma Reagent Company (Shanghai, China). C_7_–C_40_ saturated alkanes and anhydrous ethyl ether were purchased from Sinopharm Chemical Reagent Co., Ltd. (Shanghai, China). 

### 2.2. Sensory Evaluation

The tea evaluation method was slightly modified from the current Chinese national standard (GB/T 23776-2018). Briefly, 3.0 g black tea was infused at room temperature with 150 mL boiled water for 5 min. Four professional tea evaluators (aged 23–40 years with more than 3 years of sensory evaluation experience) were required to describe the quality of samples by using descriptive analysis methods and assigned scores to aroma, taste, and liquor color. According to the Chinese national standard of congou black tea (GB/T 23776-2018), the sensory scores were calculated based on 40% for aroma, 50% for taste, and 10% for soup color. Final sensory scores were expressed as the mean of three evaluations by each evaluator.

### 2.3. Quantitative Descriptive Analysis (QDA)

To investigate the aroma attributes of Yunnan congou black tea after aroma enhancement under the 90 °C condition, firstly, the panelists freely discussed and carefully selected seven representative aroma descriptors—strong, consistent, sweet, baked, caramel, fruity, and burnt—to build the aroma system of Yunnan congou black tea. All the selected aroma descriptors with high frequency of occurrence were from the sensory terms provided in the current Chinese national standard (GBT14487-2017). Then, 11 panelists (4 males and 7 females), aged between 23 and 40 years old, were trained by a series of tea aroma compounds, as shown in Table S1. The intensity of the aroma attributes was scored using a scale from 0 to 5 (where 0 = none or not perceptible intensity, 3 = moderate intensity, and 5 = high intensity). The tea brewing method was the same as that described in Section 2.2. The final score of each aroma attribute was the average value of the scores from the panelists, and was applied to render the radar diagrams.

### 2.4. Tea aroma Extraction by Simultaneous Distillation and Extraction (SDE)

The extraction method of aroma compounds was followed the modified simultaneous distillation extraction (SDE) method, described in detail by Liu et al. [14]. Briefly, 15.0 g tea sample was put in a tea vessel, and 250 mL distilled water was added in a 500 mL water drop bottle, which was placed in an electric heater at 158 °C (±2 °C). Meanwhile, 30 mL anhydrous ether was added in a 250 mL extraction bottle with a water bath at 45 °C (±2 °C). Then, the two bottles were connected and timing was started for 2 h, once the temperature rose to 100 °C. The extraction solution was collected by adding anhydrous sodium sulfate at −20 °C for 24 h, filtering, and concentrating to 5 mL.

### 2.5. Compounds Identification by GC-MS

Identification of the volatile compounds in the tea aroma extract was performed by a Shimadzu gas chromatograph 2010-plus, with a triple quadrupole mass spectrometer QP 2020 (Shimadzu, Shanghai, China). GC conditions were improved on the basis of previous studies [15]. The employed GC column was SH-Rxi-5Sil MS capillary column (30 m × 0.25 mm × 0.25 μm). The temperature of injection port was set at 250 °C in splitless mode. Helium (purity > 99.999%) was applied as the carrier gas at a constant flow rate of 1 mL/min. The oven temperature was set at 50 °C and held for 5 min, then increased to 210 °C at the rate of 3 °C/min and held for 5 min, then increased to the final temperature of 230 °C at the rate of 15 °C/min, and held for 5 min. For the mass spectrometry conditions, the ion source temperature was set to 230 °C, and the electron energy was set to 70 eV. The full scan range was 35–450 atomic mass unit. All experiments were replicated at least 3 times. The aroma components were identified based on the mass spectrum in the NIST17 library, retention indices (RI), and authentic standards. All these compounds were filtrated by the following parameter to find the valid aroma components: similarity degree > 80%, mass spectra with information from NIST 17 library at least 80%, frequency > 50%, retained entities that appeared in at least 50% of samples. The relative content of each aroma component was calculated according to the following formula: peak area of the substance/peak area of the internal standard substance (butylated hydroxytoluene) [16]. 

### 2.6. Statistical Analysis 

Correlation analysis and variance analysis were carried out by IBM SPSS Statistics 26. Aroma compounds clustering heat map was made by TBtools software, and MFA was carried out by XLSTAT 2019.

## 3. Results and Discussion

### 3.1. Sensory Evaluation of Yunnan Congou Black Tea under Different Aroma-Enhancement Conditions 

**Figure 1:**
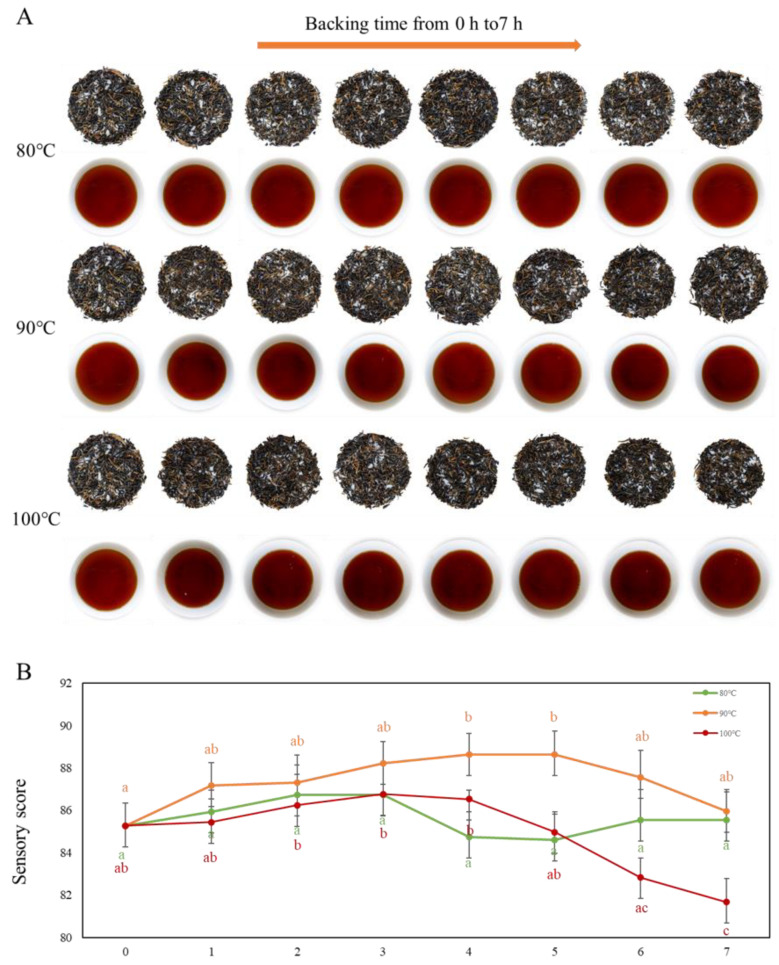
(**A**) Images of Yunnan congou black tea under different baking conditions. (**B**) The total sensory evaluation score of Yunnan congou black tea under different baking conditions of aroma enhancement. Note: Values in the same color labeled with different letters (a–c) differ significantly (*p* < 0.05).

Baking is an efficient and cost-effective method for removing the disagreeable scents and developing the beneficial flavors of tea. Different types of teas are produced with varied procedures, temperatures, and times [17,18]. When it comes to black tea, the baking process is directly associated with the development of the distinct flavor. As shown in Figure 1A, aroma enhancement treatments (baking at 80 °C, 90 °C, and 100 °C for 0–7 h) had little effect on the appearance of Yunnan congou black tea, but a higher baking temperature and duration induce a darker liquid color. The unbaked black tea sample had the following characteristics: a slightly obvious sweet aroma, a little fruity aroma, a slightly strong aroma, minty flavor, a sweet taste, a slightly strong flavor, a mellow flavor, thickness, slight umami, a brisk flavor, and orange-red and bright color (Table S2). With proper aroma-enhancement conditions, the aroma intensity was increased such as forming baked and caramel aromas, the flavor of heavy taste was increased, the color of tea liquor was bright and red, and the overall sensory score was improved. With improper aroma-enhanced conditions, the fire flavor or even a burnt flavor appeared, and the taste was slightly coarse or even bitter, decreasing the overall sensory score. These data are consistent with a previous study, showing that low-temperature baking conditions (75 °C for 3 h) gave congou black tea a redder, blooming appearance, with a brighter infusion color, stronger aroma, smoother taste, redder brewed leaves, and the highest sensory score [19]. As the quantitative data shows in Figure 1B, there was no statistically significant difference (*p* > 0.05) in the sensory scores of Yunnan congou black tea with different roasting lengths at 80 °C. However, the sensory scores showed an increase and then a decline at 90 °C and 100 °C, respectively, with a significant difference (*p* < 0.05); additionally, the sensory scores of tea samples at 90 °C were greater than the other two temperatures, with the maximum sensory score achieved at 4 h or 5 h. Upon roasting for more than 4 h at 100 °C, the sensory scores began to decline, reaching a significant level at 7 h, with the aroma displaying a fired and burnt flavor, while the taste revealed unpleasant characteristics including roughness and bitterness. Above all, in the range of roasting time from 0 to 7 h, 90 °C is the best baking temperature for the aroma enhancement of Yunnan congou black tea, leading to increased sensory score and an evident aroma enhancement impact.

### 3.2. Aroma Quantitative Descriptive Analysis (QDA) of Yunnan Congou Black Tea under Different Aroma-Enhancement Conditions

**Figure 2:**
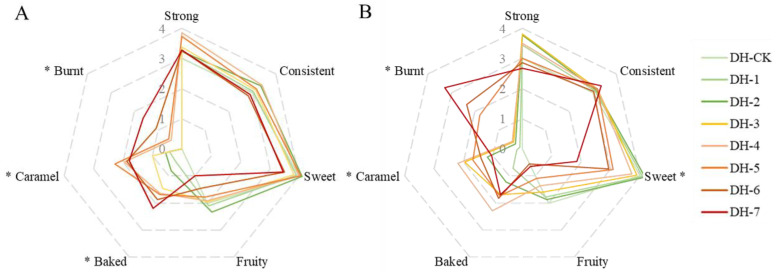
Radar diagrams of the mean sensory attributes scores of Yunnan congou black teas with different aroma-enhancement conditions. (**A**) Radar diagram of aroma-enhanced Yunnan congou black teas baked at 90 °C for 0–7 h. (**B**) Radar diagram of aroma-enhanced Yunnan congou black teas baked at 100 °C for 0–7 h. * indicates significance at *p* < 0.05. Note: DH—black tea sample of Yunnan congou black tea; DH-CK—unbaked black tea; DH-1–7—black tea baked for 1–7 h, respectively.

Because of the complexity of fragrance, a single sensory property cannot adequately express the contributions of all components. Seven representative aroma attributes were used to describe the aroma profile of Yunnan congou black tea—“strong”, “consistent”, “sweet”, “fruity”, “baked”, “caramel”, and “burnt”—where burnt was a negative descriptor, which is an unpleasant aroma attribute. As depicted in Figure S1 and Table S3, no significant differences (*p* > 0.05) in 6 aroma attributes were observed among aroma-enhanced congou black tea baked at 80 °C (0–7 h), except for the caramel aroma, which was significantly improved after baking for 7 h. There were significant differences in three aroma attributes—baked, caramel, and burnt—among the samples of congou black tea baked at 90 °C (Figure 2A). When the roasting time exceeded 3 h, the intensity of the baked and caramel aromas increased significantly. With respect to congou black tea baked at 100 °C (Figure 2B), there was a dramatic change in the aroma profile. Sweet, caramel, and burnt aromas all showed significant differences. The sensory score of the sweet aroma first increased (baking for 0–2 h), and then decreased immediately (baking for 3–7 h), finally reaching the lowest score. Tea samples baked for 3 h and 4 h both presented a significantly higher caramel aroma compared with unbaked tea. In addition, 5 h of baking produced a significant burnt flavor, which was not favorable. Among all conditions, Yunnan congou black tea baked at 90 °C for 5 h presented the highest caramel aroma, while baking at 100 °C for 4 h presented the highest baked aroma. During the baking process, the scores of sweet and fruity aromas showed a general decreasing trend, and the higher the baking temperature, the more obvious the decrease. Compared with the unbaked tea sample, the aroma strength reduced with prolonged aroma enhancement (baking at 100 °C for 6–7 h), and the aroma consistency decreased with prolonged aroma enhancement (baking at 90 °C for 6–7 h). Moreover, a higher baking temperature gave a more burnt flavor. Thus, suitable aroma-enhancement conditions were conducive to the production of the characteristic aroma attributes of Yunnan congou black tea, such as the baked and caramel aromas.

### 3.3. GC-MS Analysis of Aroma-Enhanced Yunnan Congou Black Tea Baked at 90 °C

To investigate the effect of aroma enhancement on the volatile compounds of Yunnan congou black tea, a total of 47 characteristic aroma compounds were identified by GC-MS, as shown in Table 1, including alcohols (8), aldehydes (11), ketones (8), acids (2), esters (6), nitrogen compounds (6), oxygen-containing compounds (3), and hydrocarbons (3). Alcohols were widely detected as aroma-active substances in black tea, the majority of which were produced by the decomposition of fatty acid hydroperoxides or the reduction in aldehydes [20]. Eight alcohols were identified in this study: 2-penten-1-ol, (Z)-2-furanmethanol, benzyl alcohol, linalool, phenylethyl alcohol, α-terpineol, geraniol, and (E)-3,7,11-trimethyl-1,6,10-dodecatrien-3-ol. Among these, linalool and geraniol were the most abundant (Table S4); these have also been reported as the main aroma contributors in black tea [13].

Aldehydes have lower aroma thresholds than their alcohol homologs, and hence these have a significant influence in the development of black tea scent profiles. The majority of aldehydes found in foods had green, malty, fatty, sweet, flowery, or fruity aromas, and were commonly recognized as byproducts of lipid autoxidation during manufacture. Aldehydes were the most abundant fragrance chemicals detected in this study, and the relative contents of benzeneacetaldehyde, hexanal, and (E)-2-hexenal were the largest (Table S4), which have been reported to be the major aroma-active compounds in black tea in previous research [21,22]. Ketones, with a low olfactory threshold, were mainly formed through the thermal oxidation or degradation of polyunsaturated fatty acids, amino acids, or microbial degradation, which contributed significantly to the overall aroma of tea. Eight ketones were identified, among which β-ionone was the most abundant. It has been reported as one of the key components of chestnut aroma [23].

Esters, such as methyl jasmonate (with a strong, soft, and sweet jasmine fragrance), methyl myristate (with a honey-like and iris-like aromas), benzyl benzoate (with a light, almond-like aroma), and benzyl salicylate (slightly sweet fragrance), usually contributed to fruity or floral fragrance [24]; meanwhile, some advanced fatty acids, such as methyl palmitate, methyl linoleate, and methyl linolenic acid, were reported to have a fragrance-fixing effect [25]. In the present study, six esters were detected, among which, methyl salicylate dominated, which is an important substance contributing the mint aroma and the coolness of the tea leaves [26], mainly from the hydrolysis process of glycosidic aroma substances.

The Maillard reaction and Strecker degradation have generally been confirmed to be responsible for the production of nitrogen-containing substances. N-ethylacetamide and N-ethylformamide were degraded from L-theanine, which gave large-leaf yellow tea a crispy-rice-like aroma [27], while pyridine and pyrrole derivatives produced from Strecker degradation contributed to the aged oolong tea flavor characteristics [28]. This study identified the compounds 1-ethyl-1H-pyrrole, 1-ethyl-1H-pyrrole-2-carbaldehyde, and 2-acetylpyrrole, which have previously been reported to have caramel and baked aromas.

The other aroma compounds, including acids, oxygen-containing compounds, and hydrocarbons, were also identified in this study. Among them, the dominant linalool oxygen compounds including trans-linalool oxide (furanoid) and trans-linalool oxide (pyranoid) were reported to have a positive contribution to the aroma profile of Yunnan congou black tea. In addition, styrene, an unsaturated alkane, was reported to have a burnt flavor, whereas alkanes, such as dodecane and heneicosane, contributed little to the tea aroma [29,30].

**Figure 3:**
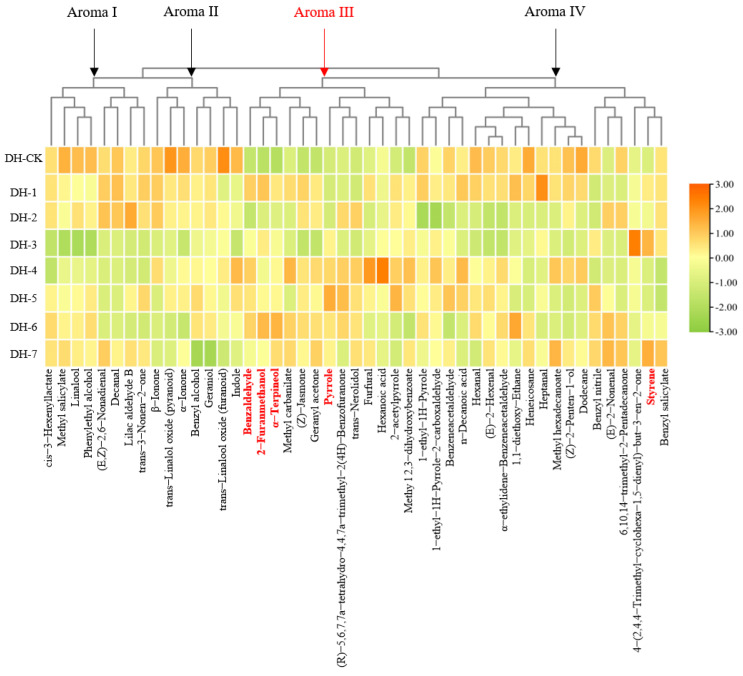
Cluster heat map of aroma compounds in aroma-enhanced Yunnan congou black teas baked at 90 °C (0–7 h).

**Figure 4:**
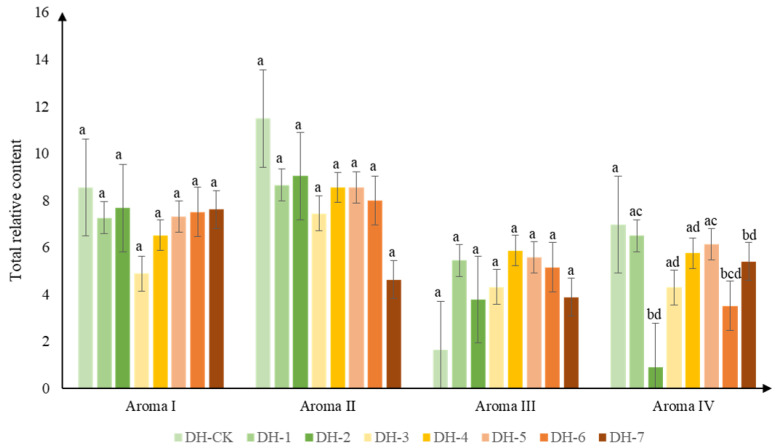
Changes of the total relative content of the four cluster classification aroma compounds in aroma-enhanced Yunnan congou black teas baked at 90 °C (0–7 h). Note: Values in each group labeled with different letters (a–d) differ significantly (*p* < 0.05).

All of the aroma compounds were divided into four groups, and each group had similar aroma characteristics, according to the aroma compounds website—http://thegoodscentscompany.com, (accessed on 15 November 2021)—or other reported research, as shown in the cluster heat map (Figure 3): aroma I (8), aroma II (7), aroma III (13), and aroma IV (19). Details of the aroma compounds descriptions are shown in Table S4. With the increase in roasting time, aroma I generally showed a decreasing trend followed by an increasing trend; aroma II appeared to have a decreasing trend; aroma III presented a growing trend followed by a decreasing trend; the total relative content of aroma IV fluctuated and was significantly lower than that of the unbaked tea sample at baking times of 2 h, 6 h, and 7 h (Figure 4). The most abundant volatile compounds in aromas I and II were linalool, geraniol, and trans-linalool oxides (furanoid and pyranoid), which commonly presented floral and fruity aromas, followed by methyl salicylate, phenethyl alcohol, and benzyl alcohol, which mainly presented sweet, fruity, and floral aromas (Table S4). Previous studies have shown that linalool, geraniol, linalool oxide, and methyl salicylate were essential aroma contributors to Yunnan congou black tea [13]. The relative contents of n-decanal (sweet and citrus aroma), benzyl alcohol (floral aroma), geraniol (floral, sweet, and citrus aromas), α-ionone (floral and woody aroma), and β-ionone (floral and woody aromas, sweet and fruity aromas) decreased significantly after baking. Aroma compounds in aroma III mainly performed caramel, baked, and woody aromas, among which 2-furanmethanol and 2-acetylpyrrole were dominant. Pyrrole (nutty, warm), benzaldehyde (caramel, almond), α-terpineol (baked, smoky, burnt), (Z)-jasmone (woody, spicy), geranyl acetone (rose, fruity), (R)-5,6,7,7a-tetrahydro-4,4,7a-trimethyl-2(4H)-benzofuranone (ripe berry, woody), and trans-nerolidol (citrus, woody) appeared to have an increased trend after baking. The relative content of 2-acetylpyrrole (baked bread, caramel) increased significantly in the middle of the baking time (4 and 5 h), and the relative content of furfural (caramel aroma, toasted bread aroma, almond aroma) also reached its highest at 4 h of baking. Therefore, the compounds clustered as aroma III were associated with the caramel and baking aromas formed in the middle and late stages of the aroma-enhancement process. Aroma IV, on the other hand, was clustered into a group of volatile compounds, mainly including phenylacetaldehyde (honey and chocolate), 1-ethyl-1H-pyrrole-2-carboxaldehyde (smoky and burnt), and hexanal (fatty, sweaty, and fruity), as well as some alkanes, such as dodecane and heneicosane, which did not contribute much to the aroma [31].

### 3.4. Multiple Factor Analysis (MFA) of Aroma Attributes and Volatile Compounds of Yunnan Congou Black Tea Baked at 90 °C

MFA is a statistical technique based on principal component analysis (PCA). Unlike standard factor analysis, which accounts for all variables together, this method is particularly well suited for cases where the same observation assesses multiple indicators, providing significant benefits for integrating and comparing data surrounding multiple features [32]. Therefore, we built a feedback platform between aroma qualities and aroma components based on MFA, in order to investigate the major aroma contributors in the aroma-enhanced black tea.

**Figure 5:**
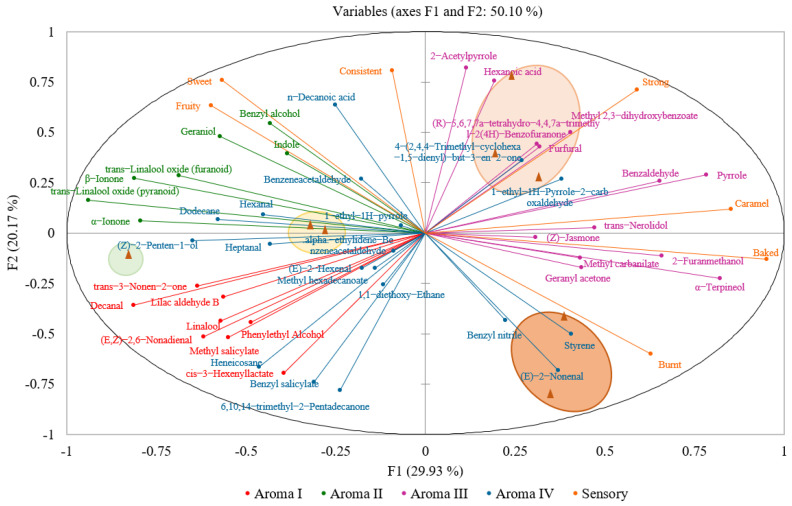
The combination of aroma attributes and aroma compounds correlation diagram and samples distribution diagram in aroma-enhanced Yunnan congou black teas baked at 90 °C (0–7 h). The green circle represents DH-CK; the yellow circle represents DH-1 and DH-2; the light-orange circle represents DH-3, DH-4, and DH-5; the orange circle represents DH-6 and DH-7. The exact location of the tea samples can be seen in Figure S3.

The first two principal components extracted were F1 (29.93%) and F2 (20.17%), with a cumulative variance contribution of 50.10% (Figure S2). As shown in Figure S3, the eight Yunnan congou black tea samples were clearly divided into four groups: DH-CK; DH-1 and DH-2; DH-3, DH-4, and DH-5; DH-6 and DH-7. Combined with the distribution map of MFA black samples (Figure 5), it was clear that the relative amounts of trans-linalool oxides (pyranoid), α-ionone, (Z)-2-penten-1-ol, dodecane, hexanal, and heptanal were the major contributors distinguishing the unroasted and short roasting period (1–2 h), aroma-enhanced black teas from the other tea samples on F1. Caramel and baked aromas and compounds of 2-acetylpyrrole, hexanoic acid, methyl 2,3-dihydroxybenzoate, (R)-5,6,7,7a-tetrahydro-4,4,7a-trimethyl-2(4H)-benzofuranone, furfural, 4-(2,4,4-Trimethyl-cyclohexa-1,5-dienyl)-but-3-en-2-one, 1-ethyl-1H-pyrrole-2-carbaldehyde, benzaldehyde, and pyrrole were distributed in the same direction as the middle roasting period (3–5 h) aroma-enhanced samples. Meanwhile, the burnt aroma, as well as 2-furanmethanol, α-terpineol, methyl carbanilate, geranyl acetone, styrene, (E)-2-nonenal, and benzyl nitrile, were distributed in the same direction as the long roasting period (6–7 h) aroma-enhanced black teas. Therefore, the above aroma attributes and compounds were likely to be important differentiations to the mid-duration and long-duration aroma-enhanced samples on F2. Additionally, the MFA results showed that the baked aroma was well correlated with the caramel aroma, and the fruity and sweet aromas were well correlated, while the relationship between the caramel and the fruity/sweet aromas showed a significant negative correlation.

Aroma I was located in the third quadrant and demonstrated a negative correlation with caramel and strong aromas, but showed a strong positive correlation with sweet and fruity aromas on F1. Aroma II appeared in the fourth quadrant and indicated a powerful positive correlation with sweet and fruity. Aroma III was found in the first and second quadrants, which exhibited a positive correlation with strong, baked, and caramel aromas. Aroma IV arose in all quadrants, suggesting that the aroma properties of this class were likely to be complex. The MFA results were consistent with prior clustering analysis. Among the individual volatile compounds, the following correlations were observed: benzyl alcohol showed a strong correlation with sweet aroma; geraniol and indole showed a strong correlation with fruity aroma; pyrrole and benzaldehyde showed a strong correlation with caramel aroma; 2-furanomethanol and α-terpineol showed a strong correlation with baked aroma; methyl 2,3-dihydroxybenzoate showed a strong correlation with strong aroma; and styrene showed a stronger correlation with burnt aroma.

Unbaked Yunnan congou black tea—normally with linalool as the main flavor component—always exhibited the primary aroma characteristics of fruity and sweet [33]. In this investigation, linalool remained the highest after 90 °C aroma enhancement, followed by linalool oxides (pyranoid and furanoid). The concentration of these aroma components was reduced with increasing roasting time, but not statistically. As can be seen, linalool and its oxides played an important role in the aromatic characteristics of Yunnan congou black tea, which could retain the primary aroma properties under proper aroma augmentation conditions. Under 90 °C aroma enhancement, the aroma changed from sweet and fruity to caramel and baked as the roasting time increased, and excessive roasting caused burnt flavor. Thermodynamic reaction during aroma enhancement process may have changed the structure and concentration of volatile chemicals or possibly created new volatile compounds. The relative concentrations of aroma substances with floral, fruity, and sweet aromas—such as benzyl alcohol and geraniol—decreased, while aroma substances with roasted and caramel aromas—such as benzaldehyde, pyrrole, and 2-acetylpyrrole—increased with increase in the roasting time. Therefore, the moderate aroma enhancement promoted characteristic aroma attributes and aroma richness in Yunnan congou black tea.

## 4. Conclusions

A sensory evaluation system/gas chromatography–mass spectrometry (SES/GC-MS) feedback platform was constructed via multiple factor analysis in this study. Appling SES, we found that the aroma characteristics of aroma-enhanced Yunnan congou black tea changed from fruity and sweet to caramel, baked, and burnt during 7 h of baking at 90 °C. Overall, baking at 90 °C for 4 h or 5 h showed the best quality-improving effect, which can increase its characteristic aroma attributes, including the caramel and baked aromas, while maintaining its primary aroma attributes, including fruity and sweet. Based on the feedback from the SES data, 47 characteristic aroma compounds of Yunnan congou black tea were identified by GC-MS, which can be clustered into 4 categories: aroma I (8) and aroma II (7) characterized aroma substances with mainly sweet, fruity, and floral aromas, including benzyl alcohol, geraniol, and indole. The contents of these substances decreased after long-duration aroma enhancement. Aroma III (13) is dominated by baked, caramel, and woody aromas, including pyrrole, benzaldehyde, 2-furanmethanol, α-terpineol, and methyl 2,3-dihydroxybenzoate, and its content tended to increase after long-duration aroma enhancement. The aroma attributes of aroma IV (19) were more complex, among which styrene was strongly correlated with burnt aroma. Excessive baking produced an undesirable aroma, which was not conducive to the maintenance of the basic aroma characteristic attributes of Yunnan congou black tea. Overall, the SES/GC-MS feedback platform can help researchers to understand the dynamic material changes causing the improved aromas to guide the baking process, as well as to establish an optional template of the relationship between the quality and aroma chemicals of teas.

## Figures and Tables

**Table 1:** Total relative contents of eight aroma compounds in aroma-enhanced Yunnan congou black tea baked at 90 °C for 0–7 h (mean ± sd, *n* = 3).

Categories	Alcohols	Aldehydes	Ketones	Acids	Esters	Nitrogenous Compounds	Oxygen-Containing Compounds	Hydrocarbons
DH-CK	11.63 ± 0.79 a	4.44 ± 0.31 a	0.39 ± 0.14 a	0.10 ± 0.06 a	1.90 ± 0.08 a	1.42 ± 0.28 a	8.05 ± 0.84 a	0.35 ± 0.26 abc
DH-1	12.48 ± 1.93 a	3.95 ± 0.31 a	0.56 ± 0.11 a	0.09 ± 0.08 a	1.75 ± 0.17 a	2.37 ± 0.39 a	5.05 ± 0.93 a	0.31 ± 0.20 abc
DH-2	9.50 ± 6.03 a	0.45 ± 0.13 c	0.62 ± 0.28 a	0.01 ± 0.01 a	1.54 ± 0.48 a	0.60 ± 0.41 a	4.11 ± 3.34 a	0.04 ± 0.01 b
DH-3	7.32 ± 3.49 a	2.31 ± 0.74 abc	0.19 ± 0.17 a	0.11 ± 0.10 a	1.19 ± 0.52 a	1.43 ± 1.15 a	4.71 ± 2.51 a	0.20 ± 0.11 abc
DH-4	10.94 ± 0.26 a	3.61 ± 0.18 a	0.51 ± 0.09 a	0.61 ± 0.53 a	1.57 ± 0.07 a	2.36 ± 0.87 a	5.97 ± 0.60 a	0.03 ± 0.02 bc
DH-5	12.14 ± 1.77 a	4.61 ± 0.51 a	0.47 ± 0.05 a	0.14 ± 0.08 a	1.50 ± 0.05 a	2.40 ± 0.71 a	5.77 ± 0.20 a	0.00 ± 0.00 c
DH-6	12.79 ± 1.74 a	1.29 ± 0.08 bd	0.37 ± 0.05 a	0.00 ± 0.00 a	1.57 ± 0.19 a	1.09 ± 0.65 a	5.52 ± 1.69 a	0.29 ± 0.21 abc
DH-7	7.11 ± 5.17 a	3.11 ± 2.07 acd	0.34 ± 0.18 a	0.00 ± 0.00 a	1.39 ± 1.06 a	1.33 ± 0.84 a	4.78 ± 2.36 a	0.47 ± 0.05 a

Note: Values in the same column labeled with different letters (a–d) differ significantly (*p* < 0.05).

## Data Availability

All data included in the main text and supplementary materials.

## Supplementary Materials

The following are available online at https://www.mdpi.com/article/10.3390/plants11060823/s1, Figure S1: Graph of the mean sensory scores of aroma enhanced Yunnan congou black teas baked at 80 °C for 0–7 h. Figure S2: Diagram of correlation between aroma properties and aroma compounds in aroma enhanced Yunnan congou black teas baked at 90 °C for 0–7 h. Figure S3: Distribution of aroma enhanced Yunnan congou black tea samples baked at 90 ℃ for 0–7 h based on MFA analysis. Table S1: Definitions, standards and reference materials used for QDA. Table S2: Sensory evaluation results of Yunnan congou black tea under different aroma enhancement conditions. Table S3: QDA scoring results of Yunnan congou black tea under different aroma enhancement conditions (mean ± sd, n = 11). Table S4: List of aroma compounds in aroma enhanced Yunnan congou black teas baked at 90 °C (0–7 h) identified by GC × MS analysis.
