# Supplementary material for: Construction of Sensory/Mass Spectrometry Feedback Platform for Seeking Aroma Contributors during the Aroma Enhancement of Congou Black Tea
Source: Plants (Basel). 2022 Mar 20;11(6):823. doi: 10.3390/plants11060823 (PMC8951376; doi:10.3390/plants11060823)
Supplement: Supplementary file 1 [file plants-11-00823-s001.zip › Figures.pdf]

Supplementary Material

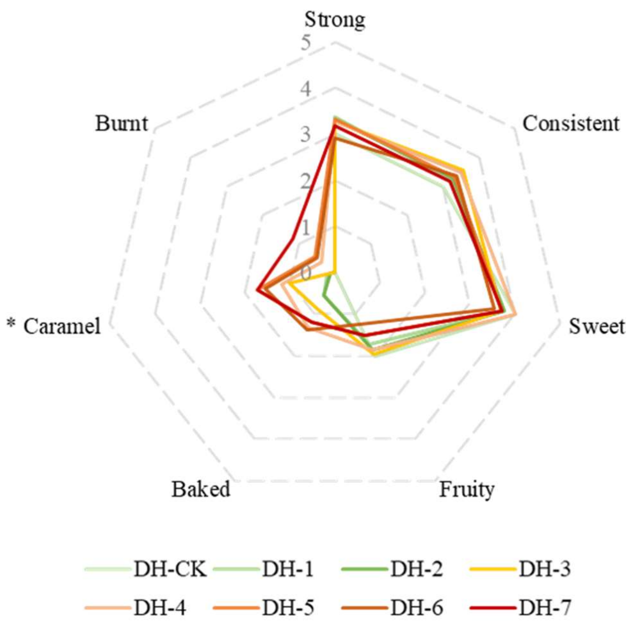

Figure S1. Graph of the mean sensory scores of aroma enhanced Yunnan congou black teas baked at 80°C for 0-7 h. * indicates significance at *P < 0.05*.

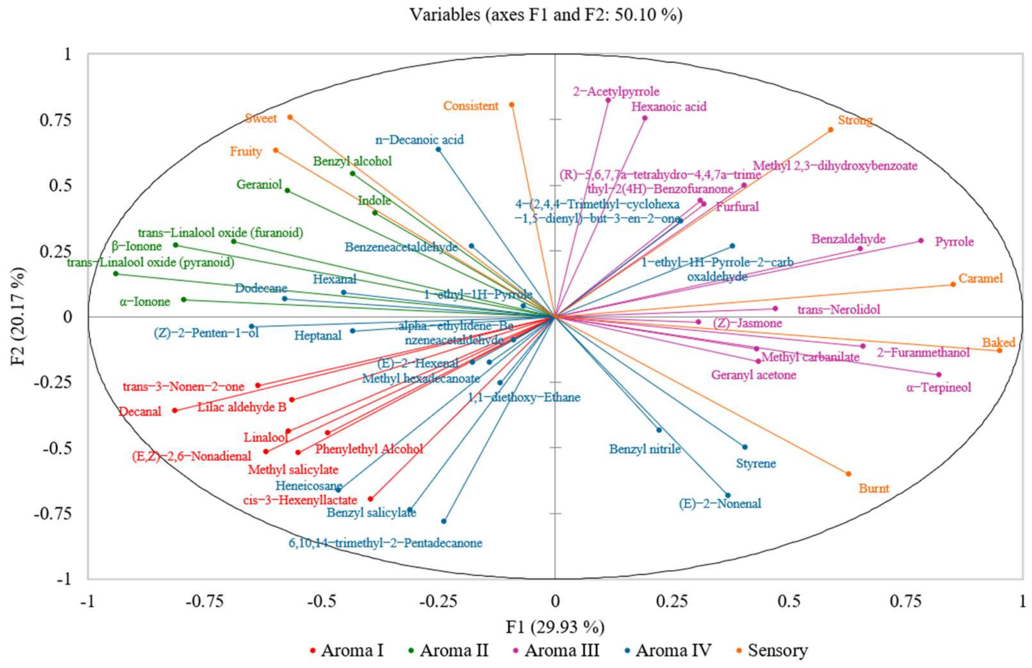

Figure S2. Diagram of correlation between aroma properties and aroma compounds in

aroma enhanced Yunnan congou black teas baked at 90°C for 0-7 h.

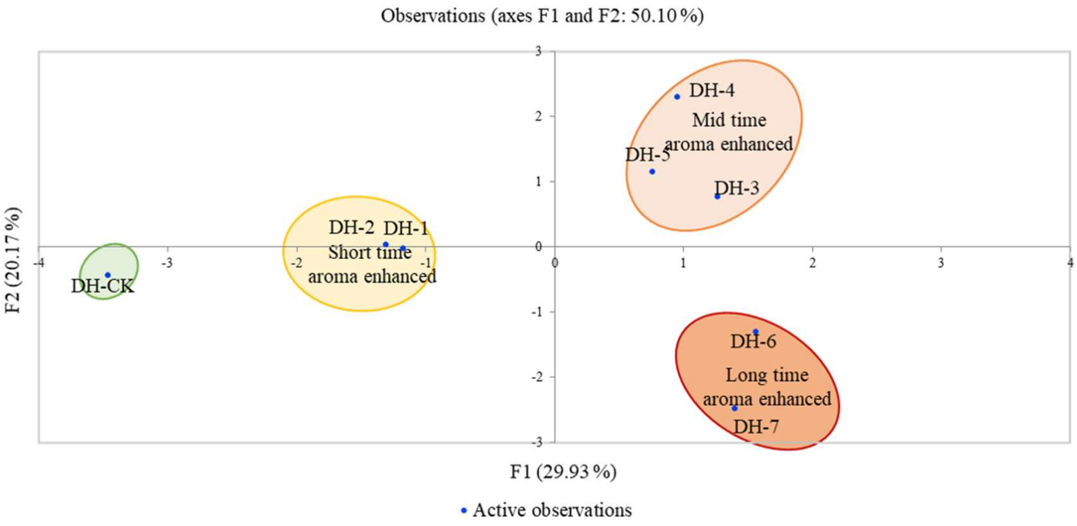

Figure S3. Distribution of aroma enhanced Yunnan congou black tea samples baked at 90°C for 0-7 h based on MFA analysis.
