# Supplementary material for: Construction of Sensory/Mass Spectrometry Feedback Platform for Seeking Aroma Contributors during the Aroma Enhancement of Congou Black Tea
Source: Plants (Basel). 2022 Mar 20;11(6):823. doi: 10.3390/plants11060823 (PMC8951376; doi:10.3390/plants11060823)
Supplement: Supplementary file 1 [file plants-11-00823-s001.zip › Tables.pdf]

Supplementary material

Table S1 Definitions, standards and reference materials used for QDA.

Attribute	Definition	Reference material	Concentration
Strong	Comprehensive intensity and richness of aroma	DH-CK	-
Consistent	The length of the fragrance retention time	DH-CK	-
Sweet	The aroma is reminiscent of sugar, such as honey, brown sugar, chocolate, etc.	Vanillin	52.70 μmol mL^{-1}
Baked	Similar to the aroma of roasted sweet potatoes and toasted bread	2,5-2 Methylpyrazine	36.25 μmol mL^{-1}
Caramel	Sweet syrup, marshmallow, aroma reminiscent of fried sugar	4-hydroxy-2,5-2-methyl-3(2H)furanone	53.54 μmol mL^{-1}
Fruity	The aroma of ripe fruits, such as orange, orange, lychee, etc.	Ethyl acetate	1.02 μmol mL^{-1}
Burnt	Unpleasant, disgusting smell, high fire smell caused by high temperature	2-methoxy- Phenol	8.68 μmol mL^{-1}

Note: The reference samples were placed in 5 mL glass bottles with lids, and the evaluator used the fan sniffing method for aroma training.

Table S2 Sensory evaluation results of Yunnan congou black tea under different aroma enhancement conditions.

Sample number	Aroma（40%）		Taste（50%）		Liquor color（10%）		
	Comments	Score	Comments	Score	Comments	Score	Total score
DH-CK	Slightly obvious sweet aroma, little fruity, slightly strong, with mint flavor	84.0	Sweet, slightly strong, mellow, thick, slight umami, brisk	85.7	Orange red, bright	88.3	85.3
DH-1[a]	Slightly obvious sweet aroma, little fruity, slightly strong, with mint flavor	84.0	Sweet, strong, mellow, thick, slight umami, brisk	87.0	Orange red, bright	88.3	85.9
DH-2[a]	Slightly obvious sweet aroma, little fruity, slightly strong, little mint flavor	86.0	Sweet, strong, mellow, thick, slight umami, brisk	87.0	Orange red, bright	88.3	86.7
DH-3[a]	Slightly obvious sweet aroma, little fruity, slightly strong, little mint flavor	86.0	Sweet, strong, mellow, thick, slight umami, brisk	87.0	Orange red, bright	88.3	86.7
DH-4[a]	Slightly obvious sweet aroma, little fruity, relatively strong	82.7	Sweet, strong, mellow, thick, slight umami, brisk	85.7	Orange red, bright	88.3	84.7
DH-5[a]	Obvious sweet aroma, little fruity, relatively strong	84.0	Sweet, strong, mellow, thick, slight umami, brisk, a little sour	84.3	Orange red, bright	88.3	84.6
DH-6[a]	Obvious sweet aroma, little fruity, with slightly firing smell and sweet potato fragrance	86.3	Sweet, strong, mellow, thick, relative umami, brisk, a little sour	84.3	Orange red, bright	88.3	85.5
DH-7[a]	Obvious sweet aroma, little fruity, with firing smell and sweet potato fragrance	86.3	Sweet, strong, mellow, thick, relative umami, brisk, a little sour	84.3	Orange red, bright	88.3	85.5
DH-1[b]	Obvious sweet aroma, little fruity, slightly strong	86.7	Sweet, strong, mellow, thick, slight umami, brisk	87.0	Heavy red, bright	90.0	87.2
DH-2[b]	Obvious sweet aroma, little fruity, slightly strong	87.0	Sweet, strong, mellow, thick, slight umami, brisk	87.0	Heavy red, bright	90.0	87.3

DH-3[b]	Obvious sweet aroma, little fruity, slightly strong, with caramel-like aroma	89.3	Sweet, strong, mellow, thick, slight umami, brisk	87.0	Heavy red, bright	90.0	88.2
DH-4[b]	Obvious sweet aroma, little fruity, strong, with caramel-like aroma	90.3	Sweet, strong, mellow, thick, slight umami, brisk	87.0	Heavy red, bright	90.0	88.6
DH-5[b]	Obvious sweet aroma, little fruity, strong, slightly obvious caramel-like aroma	92.0	Sweet, strong, mellow, thick, slight umami, brisk, a little sour	85.7	Heavy red, bright	90.0	88.6
DH-6[b]	Obvious sweet aroma, with fruity, strong, slightly obvious caramel-like aroma	91.0	Sweet, strong, mellow, thick, relative umami, brisk, a little sour	84.3	Heavy red, bright	90.0	87.6
DH-7[b]	Obvious sweet aroma, with fruity, strong, slightly obvious caramel-like aroma, with burnt aroma	87.0	Sweet, strong, mellow, thick, relative umami, brisk, slight sour	84.3	Heavy red, bright	90.0	86.0
DH-1[c]	Slightly obvious sweet aroma, little fruity, slightly strong	84.0	Sweet, slight strong, mellow, thick, slightly umami, brisk	85.7	Heavy red, bright	90.0	85.4
DH-2[c]	Slightly obvious sweet aroma, little fruity, slightly strong, with firing smell	86.0	Sweet, little strong, mellow, thick, slight umami, brisk, a little rough	85.7	Heavy red, bright	90.0	86.2
DH-3[c]	Slightly obvious sweet aroma, slightly obvious fruity, strong, with caramel-like aroma	87.3	Sweet, little strong, mellow, thick, slight umami, brisk, a little rough	85.7	Heavy red, bright	90.0	86.8
DH-4[c]	Slightly obvious sweet aroma, little fruity, strong, with caramel-like aroma	87.0	Sweet, little strong, mellow, thick, slight umami, brisk, a little rough	85.7	Heavy red, slightly bright	89.0	86.5
DH-5[c]	With sweet and fruity aroma, strong, high firing	86.0	Sweet, relatively strong, relatively mellow, relatively thick, relative umami, brisk, bitter and astringent	83.3	Heavy red, slightly bright	89.0	85.0
DH-6[c]	With sweet aroma, relatively strong, high firing	82.3	Sweet, relatively strong, relatively mellow, relatively thick, bitter and astringent	82.0	Heavy red, slightly bright	89.0	82.8
DH-7[c]	With sweet aroma, relatively strong, burnt	80.7	Sweet, relatively mellow, relatively thick, bitter and astringent	81.0	Heavy red, slightly bright	89.0	81.7

[a] aroma enhanced Yunnan congou black tea baked at 80℃. [b] aroma enhanced Yunnan congou black tea baked at 90℃. [c] aroma enhanced Yunnan congou black tea baked at 100℃.

Table S3 QDA scoring results of Yunnan congou black tea under different aroma enhancement conditions (mean ± sd, n = 11)[#].

Sample number	Aroma properties						
	Strong	Consistent	Sweet	Fruity	Baked	Caramel	Burnt
DH-CK	3.00±0.50 a	3.00±0.50 a	4.00±0.00 a	2.00±0.00 a	0.00±0.00 a	0.00±0.00 a	0.00±0.00 a
DH-1[a]	3.36±0.90 a	3.23±0.61 a	3.77±0.90 a	1.73±0.88 a	0.55±0.93 a	0.18±0.40 a	0.00±0.00 a
DH-2[a]	3.32±0.87 a	3.27±0.75 a	3.77±0.93 a	1.86±1.03 a	0.55±0.82 a	0.09±0.30 a	0.00±0.00 a
DH-3[a]	3.27±0.75 a	3.55±0.69 a	3.68±1.01 a	1.95±1.40 a	0.86±1.05 a	1.00±1.41 a	0.00±0.00 a
DH-4[a]	3.27±0.68 a	3.50±0.81 a	4.00±0.95 a	1.86±1.21 a	1.32±1.15 a	1.18±1.25 a	0.36±1.21 a
DH-5[a]	3.32±0.46 a	3.32±0.87 a	3.73±0.79 a	1.50±1.20 a	1.18±1.25 a	1.73±1.62 a	0.55±1.49 a
DH-6[a]	2.91±0.80 a	3.36±1.05 a	3.55±1.35 a	1.23±1.33 a	1.36±0.92 a	1.55±1.29 a	0.50±0.92 a
DH-7[a]	3.18±0.78 a	3.18±0.96 a	3.73±1.47 a	1.50±1.43 a	1.18±1.08 a	1.73±1.42 b	1.18±1.40 a
DH-CK	3.00±0.50 a	3.00±0.50 a	4.00±0.00 a	2.00±0.00 a	0.00±0.00 a	0.00±0.00 a	0.00±0.00 a
DH-1[b]	3.23±0.72 a	3.05±0.61 a	3.91±1.11 a	2.09±0.94 a	0.45±0.52 ac	0.41±0.66 ac	0.00±0.00 a
DH-2[b]	3.23±0.56 a	3.36±0.39 a	4.09±0.89 a	2.33±0.82 a	0.82±0.87 ab	0.55±0.69 ac	0.00±0.00 a
DH-3[b]	3.39±1.35 a	3.14±1.32 a	3.82±1.54 a	1.95±0.79 a	1.45±0.82 bc	1.00±0.72 cd	0.00±0.00 a
DH-4[b]	3.86±0.81 a	3.41±0.86 a	4.00±0.92 a	1.91±0.94 a	1.64±1.00 bc	1.95±0.79 bd	0.45±0.93 a
DH-5[b]	3.73±1.15 a	3.18±1.35 a	4.05±1.01 a	1.77±1.03 a	1.68±1.33 bc	2.27±1.03 b	0.55±1.04 a
DH-6[b]	3.27±1.27 a	2.82±1.40 a	3.50±1.28 a	1.45±1.13 a	1.86±1.52 bc	1.86±1.45 bc	1.09±1.38 a
DH-7[b]	3.27±1.54 a	2.91±1.30 a	3.45±1.29 a	1.00±1.10 a	2.18±1.17 b	1.77±1.17 bc	1.64±1.36 a
DH-CK	3.00±0.50 a	3.00±0.50 a	4.00±0.00 a	2.00±0.00 a	0.00±0.00 a	0.00±0.00 a	0.00±0.00 a
DH-1[c]	3.45±0.79 a	3.09±0.49 a	4.09±0.63 a	1.77±1.08 a	0.73±1.09 a	0.18±1.10 a	0.09±1.11 ac
DH-2[c]	3.77±0.98 a	3.14±0.50 a	4.18±0.68 a	1.86±1.19 a	1.23±1.25 a	1.18±1.17 ab	0.27±0.65 ac

DH-3 [c]	3.82±0.64 a	3.18±0.81 a	3.91±0.92 a	1.59±1.32 a	1.68±1.29 a	1.95±1.27 b	0.36±0.92 ac
DH-4 [c]	3.50±1.10 a	3.18±0.87 a	3.73±1.15 a	1.36±1.23 a	2.27±1.27 a	2.18±1.06 b	0.41±1.07 ad
DH-5 [c]	3.00±1.18 a	3.18±1.10 a	3.09±1.28 ab	1.09±1.22 a	1.73±1.27 a	1.68±1.31 ab	1.80±1.44 bcd
DH-6 [c]	2.86±1.10 a	3.05±1.06 a	2.95±1.42 ab	0.55±1.04 a	1.82±1.55 a	1.41±1.14 ab	2.36±1.55 bd
DH-7 [c]	2.68±1.19 a	3.36±1.29 a	1.86±1.34 b	0.64±1.29 a	1.68±1.59 a	1.05±1.21 ab	3.27±1.01 b

[#] Values in the same row labeled with different letters (a-c) differ significantly ($p < 0.05$).

[a] aroma enhanced Yunnan congou black tea baked at 80°C. [b] aroma enhanced Yunnan congou black tea baked at 90°C. [c] aroma enhanced Yunnan congou black tea baked at 100°C.

Table S4 List of aroma compounds in aroma enhanced Yunnan congou black teas baked at 90℃（0-7 h）identified by GC× MS analysis.

Aroma category	No.	Aroma compounds	Id [a]	Odor description [b]	Relative peak area (%)							
					DH-CK	DH-1	DH-2	DH-3	DH-4	DH-5	DH-6	DH-7
Aroma I	1	cis-3-Hexenyllactate	MS,RI	Green leafy, sweet melon, waxy, violet leaf, tropical fruity	4.20±0.03	4.10±0.01	4.10±0.01	nd	nd	3.50±0.01	4.40±0.01	4.10±0.02
	2	Methyl salicylate	MS,RI,S	Sweet, wintergreen, aromatic, camphoreous, mint flavor	168.50±0.09	147.10±0.14	142.80±0.49	110.10±0.50	136.10±0.06	142.60±0.03	148.10±0.19	155.30±0.90
	3	Linalool	MS,RI,S	Citrus, orange, floral, terpy, waxy, rose	507.50±0.57	422.20±0.13	461.50±2.69	269.40±1.81	382.90±0.08	430.00±0.09	443.30±0.53	445.40±2.57
	4	Phenylethyl alcohol	MS,RI,S	Sweet, floral, fresh bready, floral rose, honey	164.20±0.11	141.80±0.18	148.00±0.85	109.10±0.50	132.30±0.08	150.30±0.08	154.50±0.23	147.30±0.85
	5	(E,Z)-2,6-Nonadienal	MS,RI	Green fatty, dry cucumber, violet leaf	1.60±0.01	1.90±0.00	2.10±0.01	nd	nd	nd	nd	1.90±0.01
	6	Decanal	MS,RI,S	Sweet, waxy, orange peel, citrus floral	1.70±0.00	1.80±0.01	1.70±0.00	nd	nd	nd	nd	1.00±0.01
	7	Lilac aldehyde B	MS,RI	Fresh flowery	1.90±0.00	2.00±0.01	3.50±0.02	nd	nd	1.70±0.00	nd	2.20±0.01
	8	trans-3-Nonen-2-one	MS,RI	/	4.10±0.01	4.40±0.00	3.70±0.00	nd	nd	4.00±0.00	nd	3.40±0.02
Aroma II	9	β-Ionone	MS,RI	Floral, woody, sweet fruity, berry tropical, beeswax	28.10±0.16	26.20±0.04	26.60±0.15	nd	22.20±0.13	nd	nd	nd
	10	trans-Linalol oxide (pyranoid)	MS,RI	Woody	354.00±0.13	286.30±0.56	279.40±1.62	233.20±1.37	248.00±0.15	279.00±0.13	213.90±1.55	227.10±1.19

	11	α-Ionone	MS,RI,S	Violet, woody	7.00±0.04	4.90±0.00	4.00±0.02	nd	3.70±0.00	4.10±0.00	nd	3.50±0.00
	12	Benzyl alcohol	MS,RI,S	Floral, rose, balsamic	137.60±0.18	118.10±0.22	116.30±0.13	86.60±0.42	112.10±0.12	136.10±0.07	128.70±0.19	nd
	13	Geraniol	MS,RI,S	Floral, sweet, rosey, fruity and citronella-like with a citrus nuance	200.20±0.09	174.10±0.20	169.10±0.44	128.90±0.59	145.40±0.02	152.60±0.11	167.50±0.99	1.70±0.01
	14	trans-Linalool oxide (furanoid)	MS,RI	Floral	407.00±0.80	244.80±1.42	296.80±1.72	286.10±1.16	309.40±0.46	271.80±0.09	276.70±0.47	219.20±1.21
	15	Indole	MS,RI,S	Animal, floral	13.90±0.02	10.70±0.00	11.10±0.02	9.10±0.04	13.90±0.01	12.70±0.01	11.50±0.02	10.30±0.06
Aroma III	16	Benzaldehyde	MS,RI,S	Caramel, almond, fruity, powdery, nutty,	nd	17.70±0.10	nd	10.70±0.07	17.90±0.04	14.80±0.09	17.20±0.03	9.50±0.08
	17	2-Furanmethanol	MS,RI,S	Caramel, Coffee, Sweet	143.70±0.14	363.40±1.05	210.70±1.75	308.40±1.86	256.40±0.13	290.10±1.68	380.00±0.40	277.90±1.40
	18	.alpha.-Terpineol	MS,RI,S	Burnt, woody	nd	30.30±0.18	13.90±0.08	22.30±0.09	26.30±0.00	26.70±0.01	42.60±0.05	33.10±0.20
	19	Methyl carbanilate	MS,RI	/	nd	2.60±0.00	nd	nd	3.70±0.02	nd	2.90±0.02	2.70±0.02
	20	(Z)-Jasmone	MS,RI	Woody, herbal, floral, spicy, jasmin, celery	nd	14.30±0.08	12.50±0.07	nd	12.40±0.01	11.40±0.01	13.20±0.02	10.40±0.06
	21	Geranyl acetone	MS,RI	Fresh, rose, green leafy, floral magnolia, fruity	nd	5.10±0.03	5.00±0.00	nd	5.80±0.01	4.40±0.00	5.60±0.01	6.30±0.04

	22	Pyrrole	MS,RI,S	Sweet, warm, nutty, ethereal	nd	nd	nd	0.50±0.00	0.70±0.00	1.00±0.00	0.60±0.00	0.50±0.00
	23	(R)-5,6,7,7a-tetrahydro-4,4,7a-trimethyl-2(4H)-Benzofuranone	MS,RI	Ripe apricot fruity, berry, woody	nd	nd	15.50±0.09	nd	17.10±0.10	20.30±0.03	15.90±0.09	nd
	24	trans-Nerolidol	MS,RI	Floral, green, citrus, woody, waxy	nd	nd	32.50±0.06	nd	33.30±0.02	27.90±0.02	27.30±0.16	28.10±0.15
	25	Furfural	MS,RI,S	Caramel, sweet, woody, almond, fragrant baked bread	9.90±0.00	16.20±0.05	8.90±0.02	10.60±0.04	22.30±0.10	14.70±0.07	9.90±0.02	15.60±0.12
	26	Hexanoic acid	MS,RI	Sour, fatty, sweat, cheese	10.30±0.06	5.90±0.03	0.90±0.01	16.60±0.10	81.80±0.48	14.40±0.08	nd	nd
	27	2-Acetylpyrrole	MS,RI,S	Caramel-like, musty nut skin, cherry maraschino, bready	nd	84.10±0.23	73.80±0.43	58.10±0.34	102.00±0.02	125.80±0.06	nd	nd
	28	Methyl 2,3-dihydroxybenzoate	MS,RI	/	nd	3.80±0.01	3.50±0.01	3.90±0.01	5.70±0.00	4.40±0.01	nd	4.20±0.02
Aroma IV	29	1-ethyl-1H-Pyrrole	MS,RI,S	Burnt	20.90±0.02	21.40±0.02	nd	13.60±0.09	17.00±0.05	15.40±0.03	18.80±0.03	12.20±0.11
	30	1-ethyl-1H-Pyrrole-2-carboxaldehyde	MS,RI,S	Burnt roasted smoky	103.40±0.26	120.50±0.14	nd	119.20±0.71	154.50±0.89	121.00±0.70	111.40±0.65	107.30±0.67

31	Benzeneacetaldehyde	MS,RI,S	Honey, floral rose, sweet, powdery, fermented, chocolate with a slight earthy nuance	297.00±0.24	249.60±0.40	nd	186.10±0.56	235.10±0.12	330.30±0.69	nd	233.30±1.60
32	n-Decanoic acid	MS,RI	Rancid, sour, fatty, citrus	5.10±0.03	7.90±0.05	nd	nd	9.00±0.05	7.30±0.04	nd	nd
33	Hexanal	MS,RI,S	Fresh, green grass, leafy, fruity, sweaty,fatty	97.70±0.11	85.90±0.08	27.80±0.05	21.40±0.10	59.90±0.05	73.90±0.06	81.10±0.11	24.80±0.15
34	(E)-2-Hexenal	MS,RI	Green leafy	27.00±0.02	23.10±0.13	nd	nd	19.80±0.03	23.70±0.05	17.90±0.10	22.70±0.13
35	.alpha.-ethylidene-Benzeneacetaldehyde	MS,RI	Sweet, honey, cocoa, nutty, radish	5.60±0.00	5.30±0.01	nd	nd	5.50±0.00	4.40±0.00	5.50±0.01	4.10±0.01
36	1,1-diethoxy-Ethane	MS,RI	Pungent, ethereal, fruity	44.30±0.01	55.80±0.05	27.20±0.02	29.10±0.01	39.20±0.02	25.90±0.03	61.70±0.05	31.30±0.04
37	Heneicosane	MS,RI	Waxy	44.40±0.26	30.40±0.18	nd	6.00±0.04	nd	nd	26.20±0.22	28.10±0.13
38	Heptanal	MS,RI	Fresh, fatty, green herbal	3.10±0.02	6.20±0.01	nd	2.20±0.01	0.40±0.00	1.70±0.01	nd	1.70±0.01
39	Methyl palmitate	MS,RI	Oily, waxy, fatty	13.80±0.01	13.70±0.01	nd	nd	18.50±0.11	nd	nd	21.30±0.08
40	(Z)-2-Penten-1-ol	MS,RI,S	/	9.30±0.01	7.90±0.02	5.70±0.05	nd	7.90±0.05	nd	nd	6.30±0.05
41	Dodecane	MS,RI	Odorless	5.10±0.00	3.20±0.02	nd	nd	3.80±0.02	nd	nd	2.00±0.01

42	Benzyl nitrile	MS,RI	/	3.60±0.01	nd	nd	3.00±0.02	nd	4.10±0.00	3.60±0.01	3.70±0.02
43	(E)-2-Nonenal	MS,RI	Fatty, green cucumber, citrus	nd	nd	4.30±0.03	nd	nd	2.20±0.01	4.90±0.03	5.40±0.03
44	6,10,14-trimethyl-2-Pentadecanone	MS,RI	/	11.20±0.00	nd	11.50±0.02	nd	nd	nd	11.20±0.06	12.60±0.06
45	4-(2,4,4-Trimethyl-cyclohexa-1,5-dienyl)-but-3-en-2-one	MS,RI	/	nd	4.20±0.02	4.40±0.03	28.00±0.17	3.90±0.02	3.80±0.02	nd	nd
46	Styrene	MS,RI,S	Sweet, balsamic, floral, plastic, almond	nd	8.80±0.01	4.30±0.01	15.90±0.10	nd	nd	4.90±0.03	16.50±0.10
47	Benzyl salicylate	MS,RI	Balsamic, clean herbal, oily, sweet	5.10±0.00	5.10±0.03	5.20±0.03	5.00±0.02	nd	nd	3.40±0.02	6.50±0.05

[a], identification method. MS, identification based on the NIST 2017 mass spectral database; RI, retention index; S, the compounds were identified using authentic standard compounds. [b], the aroma descriptions of the aroma compounds in the table were all from the aroma compounds website: http://thegoodscentscompany.com; /, [c], relative peak area represented relative content which was expressed as mean ± sd; aroma descriptions are not found; 'nd', aroma compounds were not detected.
